# The Neighborhood as a Unit of Change for Health: Early Findings from the East Harlem Neighborhood Health Action Center

**DOI:** 10.1007/s10900-019-00712-y

**Published:** 2019-08-26

**Authors:** Rachel Dannefer, Barbara C. Wong, Padmore John, Jaime Gutierrez, La’Shawn Brown-Dudley, Kim Freeman, Calpurnyia Roberts, Elana Martins, Ewel Napier, Philip Noyes, Hannah Seoh, Jane Bedell, Cassiopeia Toner, Torian Easterling, Javier Lopez, Noel Manyindo, Karen Aletha Maybank

**Affiliations:** 1 Harlem Neighborhood Health Action Centers, Center for Health Equity, NYC Department of Health and Mental Hygiene, 161-169 East 110th Street, New York, NY 10029 USA; 2 Division Management, Center for Health Equity, NYC Department of Health and Mental Hygiene, Long Island City, NY USA; 3 Bronx Neighborhood Health Action Centers, Center for Health Equity, NYC Department of Health and Mental Hygiene, Bronx, NY USA; 4 Brooklyn Neighborhood Health Action Centers, Center for Health Equity, NYC Department of Health and Mental Hygiene, Brooklyn, NY USA; 5 Systems Partnerships, Center for Health Equity, NYC Department of Health and Mental Hygiene, Long Island City, NY USA; 6 Center for Health Equity, NYC Department of Health and Mental Hygiene, Long Island City, NY USA

**Keywords:** Health equity, Place-based interventions, Collaboration, Local public health departments, Service co-location

## Abstract

Place-based approaches have been promoted as one way to reduce health inequities by addressing community-level factors that shape health, such as housing quality, healthcare systems, the built environment, and social capital. In 2016–2017, the NYC Health Department’s Center for Health Equity launched three Neighborhood Health Action Centers (Action Centers), which use a place-based approach to improve health in neighborhoods with disproportionate burdens of premature mortality. We describe this approach and the genesis of the Action Centers. We then describe the East Harlem Action Center, which was the first to open, and share findings from qualitative interviews with the East Harlem Action Center’s Governance Council, a group comprised of Action Center staff and co-located partners and programs which supports Action Center coordination. Interviewees felt that collaboration, being responsive to community needs, and being community based were essential elements of the Action Center. Interviewees recognized the complex dynamic of a large city agency serving as the host for the Action Center while simultaneously aiming to establish more equitable relationships with partners. Governance Council members’ expectations and hopes for the East Harlem Action Center were consistent with the overall vision and model for the Action Centers, which may facilitate implementation.

## Introduction

Though New York City (NYC) is one of the wealthiest cities in the United States, it is also one of the most economically unequal and racially segregated. Since the colonization of NYC in the 1600’s, racially-based discriminatory practices have directed where people live and the resources available in their neighborhoods [1]. The cumulative impact of these processes has led to stark differences between neighborhoods based on race and wealth, affecting issues such as housing conditions and public resources and contributing to deep and persistent health inequities.

**Fig. 1:**
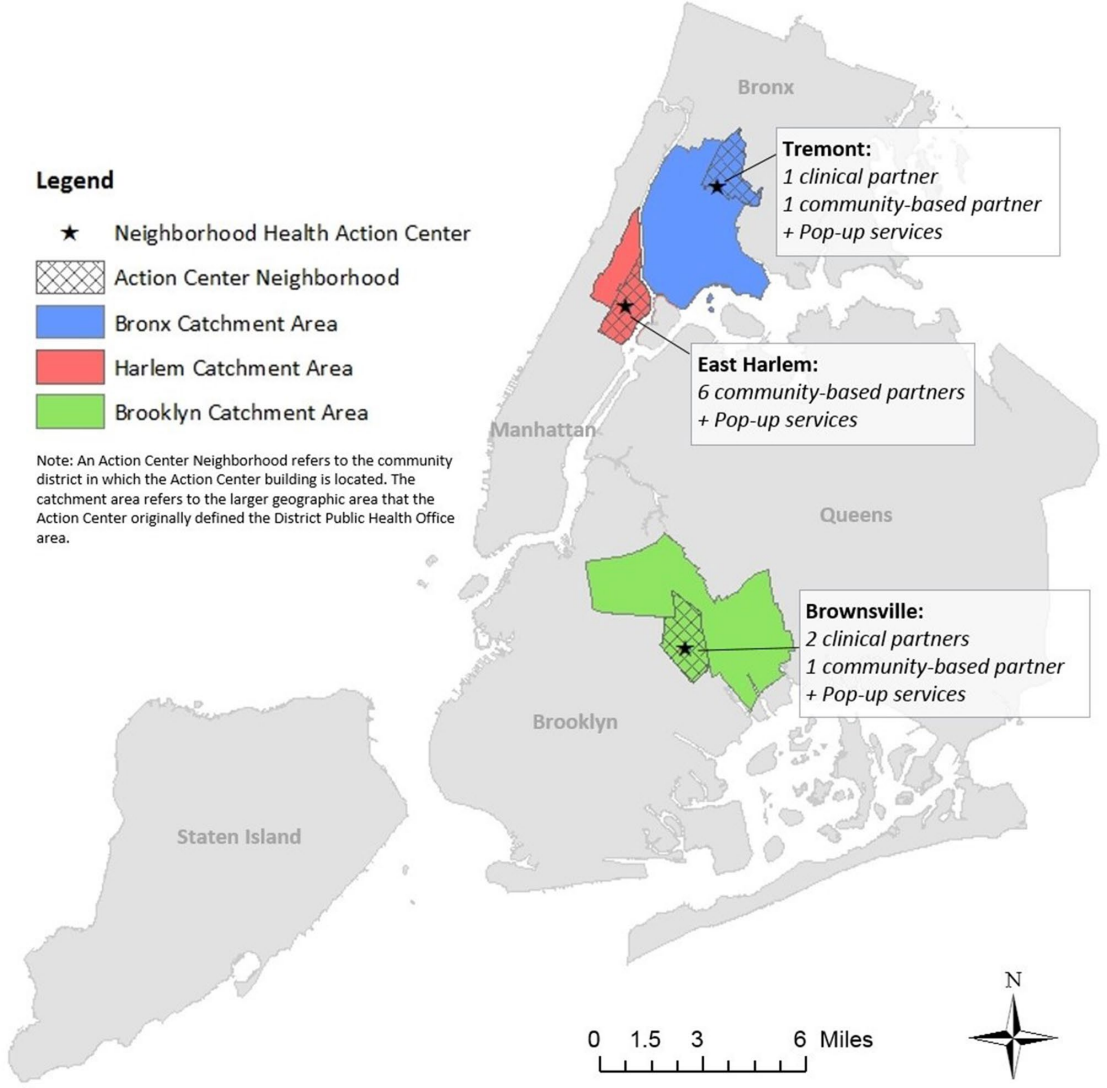
NYC Health Department Neighborhood Health Action Centers, 2017

Place-based approaches have been promoted as a way to reduce health inequities by addressing community-level factors that shape health, such as housing quality, healthcare systems, the built environment, and social capital [2]. In 2016–2017, the Center for Health Equity at the NYC Department of Health and Mental Hygiene (Health Department) launched three Neighborhood Health Action Centers (Action Centers), which use a place-based approach to improve health in neighborhoods with disproportionate burdens of premature mortality (Fig. 1). The Action Centers are part of a neighborhood strategy that co-locates clinical and community-based services, leverages a referral system to facilitate linkages across service providers and meet residents’ social and health needs, offers a vibrant community space and local programming, and seeks to engage communities and amplify community power to bring the lived experiences and input of marginalized groups to greater prominence to affect policy and systems change. Recognizing neighborhoods as a key unit of social transformation, Action Centers aim to address interrelated forms of oppression such as racism, inequitable wealth distribution, differential access to community power and resources, and the resulting poor health outcomes with the long-term goal of eliminating inequities in infant and premature mortality and self-reported health.

The Action Centers bring together a number of evidence-based approaches to improve health. Co-location of community-based resources, multi-disciplinary services, and development of referral systems have been identified as effective strategies to address social determinants of health, provide preventive services, and improve health outcomes [3, 4]. Interventions employing broad-based community collaboration and partnerships have also been effective in addressing social determinants of health and improving community health [5]. However, to our knowledge, the Action Centers are the first instance of a local health department bringing together co-location, a referral and linkage system, coordination and community engagement, and efforts to amplify community power for a place-based approach to improve neighborhood health.

In this paper, we discuss our neighborhood strategy and the genesis of the Action Centers. We then describe the East Harlem Action Center, which was the first to open, and share findings from interviews with its Governance Council, which supports coordination for the Action Centers. These interviews explored early expectations for and implementation of the East Harlem Action Center.

## Background

### The Genesis of the Action Centers

The Action Centers build on the legacy of NYC’s District Health Center movement, which began in 1921 with a pilot project in East Harlem to co-locate health services, welfare agencies and community-based organizations to coordinate health and social services for the neighborhood [6]. Based on this project’s success, District Health Centers were replicated in other NYC neighborhoods until World War II and other events led to their defunding as resources went elsewhere. In 2003, under then-Deputy Commissioner Dr. Mary T. Bassett, the Health Department renewed its neighborhood focus by establishing District Public Health Offices. These offices promoted health equity through resources, programming and partnerships in NYC neighborhoods with the highest rates of illness and premature death: the South Bronx, East and Central Harlem, and North and Central Brooklyn.

In 2014, Dr. Bassett was appointed Health Commissioner for NYC, and under her leadership advancing health equity became a clearly stated agency goal. As part of this commitment to health equity, vacant or underutilized Health Department buildings in the District Public Health Office neighborhoods were selected to become Action Centers, serving as a way to reinvest in marginalized neighborhoods. The Action Centers would expand on the work of the District Public Health Offices and represent a new way to work with local communities, prioritizing community expertise and establishing mechanisms for residents and organizations to inform Health Department efforts to improve neighborhood health. The Action Centers would provide low-cost office space to co-locate partner organizations, allowing residents to access a broader range of services than the Health Department could offer alone. They would also offer free convening space, host community events, feature common spaces to foster cross-sector interaction, and offer a wide spectrum of programs—from art exhibitions to legal services—to promote health. The Action Centers have two geographic boundaries. The Action Center “neighborhood” is the community district in which the Action Center building is located, while the “catchment area” includes additional community districts that defined the District Public Health Office areas (Fig. 1).

### Neighborhood Strategy

The Action Centers are part of a neighborhood strategy that is informed by reimagining the neighborhood health center movement that started in East Harlem in 1921, and more contemporarily by the Bay Area Regional Health Inequities Initiative framework, which illustrates the connections between health and social inequalities [6, 7]. The framework encourages the public health community to shift from its traditionally “downstream” focus on individual behaviors to focus on the “upstream” factors of social and institutional inequities and living conditions [7]. The Action Centers were also inspired by other models of co-location, including the settlement house movement, and by the Community-Centered Health Home model [8, 9].

**Fig. 2:**
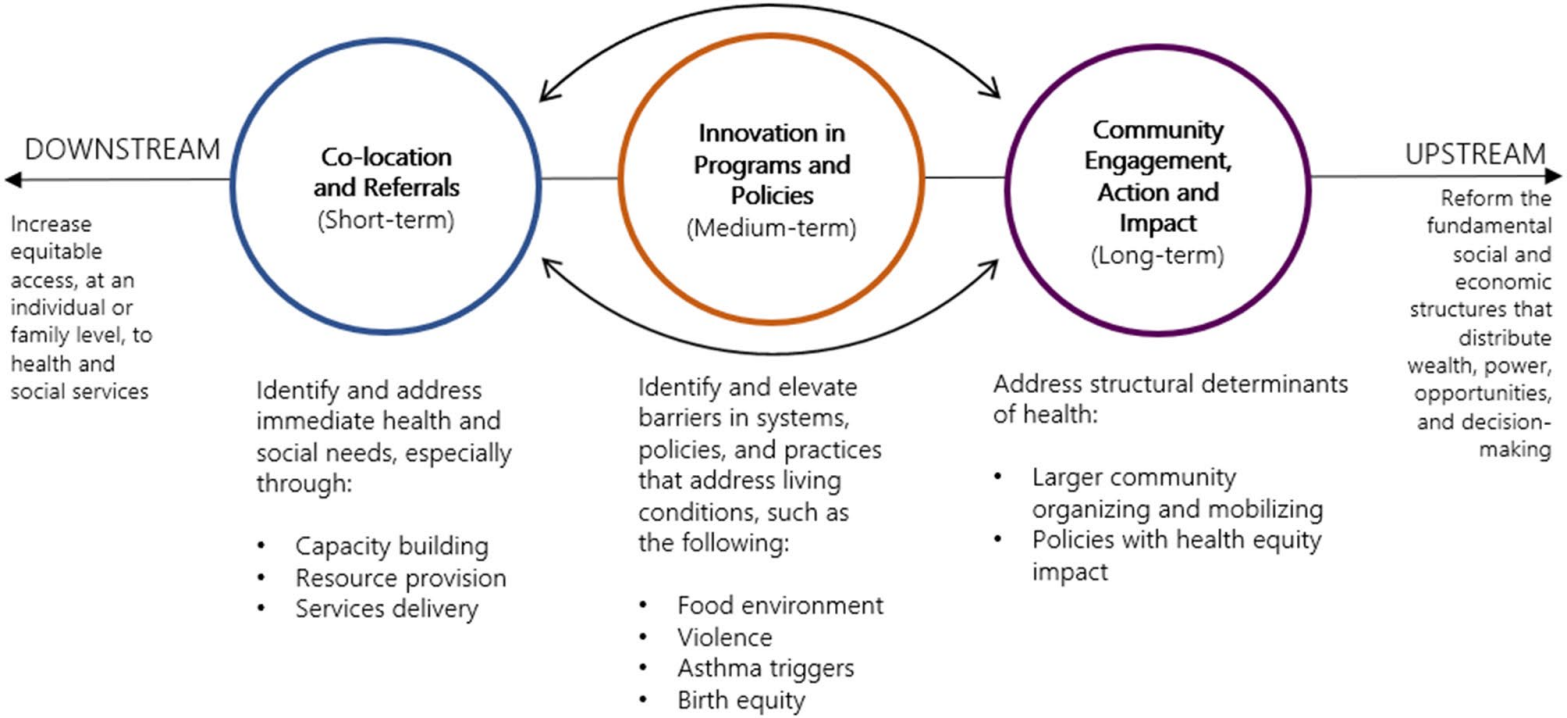
Neighborhood strategy components for the NYC Health Department Neighborhood Health Action Centers

The neighborhood strategy is comprised of three components to address social and institutional issues that affect health (Fig. 2). The first is co-location of services and a referral system to better serve residents, coordinate services, and identify gaps in coverage and reduce duplication. The second is innovation in programs and policies by using data and resident expertise to ensure that programs, systems, and policies are responsive to neighborhood needs and gaps, and to leverage the Health Department’s power to coordinate with other city agencies to improve neighborhood conditions. The third is community engagement, action and impact through collaboration with community-based organizations and residents, with the goal of bringing community voices to city agency decisions around resources and policies. These components are mutually reinforcing and reflect the intention to build on downstream, individually-focused strategies to support the upstream work of collective action and impact. These components are operationalized through several programmatic features (Table 1).

**Table 1:** NYC Health Department Neighborhood Health Action Centers, features and structure

Building activation	Action Centers have worked to create attractive, welcoming spaces, for example removing security bars from windows and partnering with local artists to feature art within the buildings. Action Centers offer free meeting space to community-based organizations and residents.
Co-located partners	Co-located partners are selected through a Request for Proposal process and pay reduced fees for their office space. Co-located partners offer services such as primary care, mental health care, youth programming, and benefits enrollment. Action Centers host pop-up services from local organizations to complement existing services and programs.
Health Department programs	The Action Centers feature a range of neighborhood-based Health Department programs addressing issues such as childhood asthma, birth equity, teen pregnancy, and physical activity and nutrition.
Family Wellness Suite	Located in each Action Center and feature a lactation lounge and children’s nook. Offers programs to improve women’s health by addressing toxic stress and increasing access to reproductive health, and programs to increase infant safety.
Referral and linkage system	Action Center staff provide referrals to neighborhood services using NowPow, an electronic referral system and resource directory that will allow for bi-directional referrals between the Health Department and partners.
Governance Council	Comprised of Action Center staff, co-located partner leadership (and in some sites other local partners), and co-located Health Department programs. Supports coordination of services and responsiveness to community needs. Monthly meetings allow partners to exchange information and identify ways to work together, and feature presentations from local organizations to facilitate collaboration and referrals beyond building partners.
Outreach and promotion	Outreach strategies include visiting local organizations and businesses, presenting to local coalitions, hosting open houses and building tours, and media campaigns.
Research and evaluation	Research and evaluation staff from the Health Department support efforts to collect process data and to understand the impact of the Action Centers; efforts are made to share findings with co-located partners.

**Table 2:** East Harlem Neighborhood Health Action Center co-located partners and programs, NYC, 2017

*Co-located partner organizations*	
Association to Benefit Children—Fast Break Mental Health Program	Provides psychiatric evaluations and ongoing mental health treatment to children, adolescents and their families.
Concrete Safaris	Prepares youth to lead healthy lives and shape their environment through outdoor education, play, exploration and community engagement.
IDNYC	Offers a photo identification card for all residents of New York City age 10 and older. This municipal ID card connects New Yorkers to services, programs, and benefits, regardless of immigration status, homeless status, or gender identity.
Manhattan HIV Care Network	Provides gender friendly, culturally appropriate referrals and information about HIV and hepatitis C testing, prevention, housing, legal services, back to work opportunities, food pantries, linkage to care, insurance, HIV research and additional resources.
Public Health Solutions	Helps individuals and families apply for free or low-cost health insurance coverage and the Supplemental Nutrition Assistance Program (SNAP, also known as Food Stamps).
SMART University: Sisterhood Mobilized for AIDS/HIV Research & Treatment	Provides the latest treatment, prevention, nutrition and health education in a supportive peer community, so that women, trans women and youth living with or affected by HIV/AIDS can enjoy longer, healthier, and more productive lives. SMART focuses on a “food as treatment” model to empower low-income New Yorkers to learn healthy cooking and eating habits and to have access to high-quality affordable foods.
*Selection of co-located Health Department programs*	
East Harlem Asthma Center of Excellence	Offers free services for families to help control asthma and improve overall health. Asthma counselors provide self-management tools to children and their caregivers, connect them to pest control, provide educational workshops and referrals, and host fun children’s events.^a^
Eat Healthy—Live Life	Offers nutrition workshops in English, Spanish, and Mandarin, with physical activity and food demonstrations for healthy eating. Participants receive Health Bucks for shopping at farmers' markets throughout the city.
Harlem Health Advocacy Partners	Helps residents of public housing and other community members access free health coaching, insurance navigation, wellness activities, and community mobilization around health and housing.^b^
Pest Control Services	Conducts pest management activities to prevent the transmission of rodent-borne diseases, prevent rodent bites, and improve the quality of life for New York City residents by reducing commensal rodent populations.
*Pop-up services (offered regularly at the Action Center by organizations based in other locations)*	
Harlem United Dental Van	A mobile dental van providing low-cost dental services to both insured and uninsured individuals, regardless of ability to pay. Referrals to non-general dental services are provided.
Harlem United Medical Van	A mobile medical van offering primary / preventive services to all regardless of ability to pay.
Lenox Hill Neighborhood House	Offers free, comprehensive civil legal services for housing using a multi-disciplinary holistic approach to legal representation.

^a^Ref. [10]

^b^Ref. [11]

## Methods

### The East Harlem Action Center

The first Action Center to open was in East Harlem, which is currently home to 124,323 people and is 50% Latino, 30% Black, 12% White and 6% Asian [12]. East Harlem is a vibrant neighborhood with a history of offering refuge to oppressed groups, local organizing, and significant cultural contributions [13]. Like many neighborhoods of color, East Harlem has been subject to racist policies of residential segregation and municipal disinvestment, negatively impacting conditions for health [14]. Life expectancy in East Harlem is almost a decade lower than that of the adjacent, wealthier Upper East Side neighborhood, and rates of infant and premature mortality in East Harlem are higher than citywide rates [12, 15].

The East Harlem Action Center launched in September 2016, bringing new life to the building that served as the original District Health Center in 1921 and which subsequently housed various health clinics. Before its launch as an Action Center, local community groups had requested that the building be revitalized in a manner responsive to community needs. Today, the East Harlem Action Center has six co-located partners and space that will be used by a clinical provider. Partners provide behavioral health services for children, benefits enrollment, youth programming, nutrition and wellness programming, free NYC identification cards, and more (Table 2). The East Harlem Action Center also houses co-located Health Department programs and hosts pop-up services, such as legal services and a mobile dental clinic. In its first year, the East Harlem Action Center received more than 15,000 visitors and made 600 referrals for health and social needs.

Like all Action Centers, the East Harlem Action Center has a Governance Council comprised of Action Center staff, co-located partner leadership, and co-located Health Department programs. The Governance Council meets monthly to promote service coordination, foster transparency, address building issues, and ensure responsiveness to community needs.

### Governance Council Interviews

In early 2017, soon after the East Harlem Action Center opened, we conducted qualitative interviews with its Governance Council members. Interviewees were asked to describe the Governance Council and to share their expectations of and early experiences with the council and Action Center, and recommendations. We aimed to document the process of Governance Council formation, provide early feedback to Action Center staff, and establish a baseline to be compared to future interviews with Governance Council members. This project was reviewed by the NYC Health Department’s Institutional Review Board and determined to be exempt human subjects research.

We interviewed 10 Governance Council members including 2 Action Center staff, representatives from 4 of the 6 co-located partners, and a representative from each of the 4 Health Department programs in the building. Co-located partner and program representatives were from leadership roles. The co-located partner organizations represented were diverse, ranging from large public health organizations with decades of experience to smaller East Harlem-based non-profit organizations which did not have permanent office space until moving into the Action Center. Interviewees attended at least two Governance Council meetings prior to being interviewed. Verbal consent was obtained at the beginning of the interviews. Interviews were audio-recorded, transcribed, and analyzed using the constant comparison method and thematic analysis [16, 17]. Transcripts were coded in Atlas.ti (version 7.0, Berlin, Germany, 2012).

## Results

Below, we present key themes from the Governance Council interviews related to Action Center development and Governance Council formation.

### Collaboration and Co-location

Governance Council members consistently spoke about collaboration as an essential element and important benefit of the Action Center model and shared ways that partners could collaborate:

As we all get to know each other in the building and…we're working together and with referrals, working on maybe even health fairs in the building, just different initiatives so people know we're in tune with each other. -Co-located partner

I'm excited that we're going to be collaborating with other people, that they're going to be right down the hall. I think that's going to be very interesting. -Co-located partner

Governance Council members also expressed excitement about co-location and referrals and saw value in their clients being able to access multiple services within the building. Some emphasized the importance of eventually having a clinical provider in the building.

We're small…So we don't do a lot of the services…that are offered here from other agencies. So it would be good…if we can refer some of our clients to different places here…I think eventually we'll really be able to say, oh, you know what, and we can send you over there… -Co-located Partner

Monthly Governance Council meetings were identified as an important mechanism for learning about partners and fostering collaboration and coordination.

We come from different perspectives and agencies and, you know, we're given that, forum to be able to talk about what our needs may be and how we can work together… [At Governance Council meetings] I get to know who's in the building with us and what they do and how we can collaborate… -Co-located Partner

Interviewees identified other methods to support collaboration, including one-pagers about the partners, orientation for new members, and shared screening questions to facilitate referrals.

### Being Neighborhood-Based

Being neighborhood-based and responsive to community needs was a commonly recognized part of the Action Center model and a point of unity across interviewees. Some discussed the importance of community members recognizing the Action Center as a trusted resource and building on its history in the community.

I think [our staff are] excited to be more community-based…and to have it be like part of a network…and be part of something kind of bigger than just our organization. -Co-located partner

I know that a lot of the older people in the community, when you say to them, I'm working out of the building on 115th—they're like, wow, I used to go there to get my birth control, or I used to go there for my checkups, I took my children there. So they knew this as the health center—and that they can go there for assistance. And I'm hoping to get that. -Health Department Program Staff

Governance Council meetings were seen as a place to identify gaps and coordinate to meet community needs and provide high-quality services.

I’d like to see … where one portion of the meeting would look at just data, like hard cold data, like productivity and outreach and…with that data analyze it and strategize where, how each agency would… better fit into the community. -Co-located partner

I would like to see where we have very robust conversations about how we can improve services… We recognize that we can't do everything, that there are gaps in what we can provide, let's find the other organizations that can help us…because we want to have a complete service solution to our clientele. –Action Center staff

### Relationship between the Health Department and Partners

The relationship between the Health Department and co-located partners was a common theme. Partner reflected on the experience of forging a new type of relationship with the Health Department. One partner observed that being part of the Action Center was a “different relationship with the Health Department” than they had experienced before:

I have been in meetings where you sit around and everyone is like, the big boss is there. You guys are—it's different, because it's a different relationship. I don't think it will turn out that way but I've been to like contract meetings at DOH.… all the partners are in the rooms…and nobody says anything about what's really because everyone was like…if I say that, am I going to get in trouble? -Co-located Partner

Partners commented that Action Center staff were more inclusive and transparent, including through the tone set for Governance Council meetings.

People know that it’s political and that there are bureaucracies, and again, I like the fact that [Health Department staff has] just been so transparent…I think follow up, just constant, we know you’re here, we know your ideas, we’re working on it…. that goes a long way. Rather than just not telling us anything… –Co-located partner

I like the fact that there are different levels, level of staff participating in [the Governance Council], because we see the different perspectives. I like the fact that there is that democracy where people have that freedom of speech and, and each idea is respected. -Co-located partner

Others noted that there was still a power dynamic between the Health Department and partners. One interviewee questioned whether the Governance Council had a leadership role or an advisory role, noting, “Ultimately the Department of Health is really in charge.” Other comments spoke to the challenges presented by the bureaucracy inherent in working with large governmental agencies.

Working with any government agency takes a really long time to get anything done and I don't think the intention is not to be transparent but it's very difficult to plan around things that seem like they may be happening someday. – Co-located partner

Action Center staff recognized the need for the Health Department to work with partners—including partners outside of the building—to be effective.

I think it is really important that we realize that there's a limitation even to the strengths of…an organization this big—that we cannot do it alone, we have to collaborate. -Action Center Staff

Action Center staff also spoke about the need for respect and humility as the host for the Action Center. They expressed a desire to set collective goals and to be informed by community expertise.

Well, I hope…to have the larger vision of what we want to accomplish and to continue pushing all the organizations along towards that larger goal. And that goal is not necessarily a DOHMH goal. I think the goal is larger… Some of these organizations that have been doing their work… for quite some time, they've done a great job at it…they've been addressing [their goals] in a fantastic way. So, it's not necessarily to say that DOHMH has a large overarching goal that supersedes anybody else’s, but how can all of our goals be melded into one, so we have a common vision and a common goal. -Action Center Staff

We can chart a path forward and make sure that you guys [community organizations/representatives] are providing input because we look to you as experts of the community. -Action Center Staff

There's a benefit to seeing how a lot of organizations that have been in the community, how they worked with those clientele, with those community residents. So there's a lot that we can learn from our community partners. -Action Center Staff

Governance Council meetings were seen as a critical avenue for ensuring open communication between the Health Department and partners.

Those meetings are going to be really important. … You just want people to be able to say what's really happening, right? Like going forward, you want people to be able to like actually openly share any concerns they have and not feel like they're going to be, you know, in trouble or get their head bit off or something if they like share that there's a problem. -Co-located Partner

We are working better together by having these monthly meetings where we get to discuss things that are working well, not working well and new things that are coming…My expectation is that it becomes a place where conflict gets resolved if there's any, that it becomes a trust, trustworthy kind of place or body to address difficult or challenging situations. -Action Center staff

## Discussion

In this paper, we describe a health department-led place-based intervention which uses a health equity approach and we share early experiences from the East Harlem Action Center. Governance Council members in East Harlem agreed on the importance of collaboration for the Action Center and on the benefit of being both community based and responsive to community needs. Interviewees also recognized the complex dynamic of a large city agency serving as the backbone for the Action Center while simultaneously aiming to establish more equitable and responsive relationships with partners. Co-located partners noted that the Action Center represented a new type of relationship with the Health Department, while staff discussed working with partners to set collective goals and highlighted the importance of community expertise and input. This perspective aligns with the Action Centers’ goal of establishing new ways for organizations and residents to shape the Health Department’s neighborhood work. Taken together, these findings demonstrate that Governance Council members’ expectations and hopes for the East Harlem Action Center were consistent with the overall vision and model for Action Centers, which may facilitate implementation. As these partnerships deepen, they will be key for collective efforts to affect upstream policy and systems change.

The Action Centers invest in neighborhoods that have historically endured disinvestment while bringing together a number of approaches to improve health. While many examples of co-location have demonstrated benefits for organizations and clients, to our knowledge, the Action Centers are unique in that they are led by the Health Department and repurpose city-owned buildings to serve as a resource for local community organizations and residents [18]. The Action Centers complement other models for collaboration between local health departments and communities, some of which have produced programmatic, policy and systems changes that can be expected to yield long-term improvements in community health [5]. The Action Centers aim to bring these components together to move efforts upstream and advance health equity by creating space for resident-led advocacy and community leadership to influence policy and systems change.

In addition to the East Harlem Action Center, the Tremont Action Center in the Bronx and the Brownsville Action Center in Brooklyn are open and active. All Action Centers have a Governance Council and are finding ways to be a community resource. For example, all have featured art exhibits and host pop-up organizations to expand services within the building. An electronic referral system is being rolled out across the Action Centers which links the Health Department, co-located partners, and other community partners, and Action Centers are also developing resident-led committees to inform their work. Finally, evaluation of the Action Centers is ongoing, with current efforts focusing on monitoring process measures and developing mechanisms to evaluate Action Centers’ impact on neighborhood health and health inequities.

Place-based initiatives are increasingly being promoted to address social determinants of health and health inequities, with public health leaders recommending place-based work and multilevel approaches to achieve health equity [19, 20]. Through the Action Centers, we offer one example of a place-based approach and share experiences from early stages of implementation. This model demonstrates a way to approach the neighborhood as a unit of change and to leverage diverse assets within a neighborhood to achieve improvements.
